# Nanoencapsulation of ABT-737 and camptothecin enhances their clinical potential through synergistic antitumor effects and reduction of systemic toxicity

**DOI:** 10.1038/cddis.2014.413

**Published:** 2014-10-09

**Authors:** D Schmid, G E Jarvis, F Fay, D M Small, M K Greene, J Majkut, S Spence, K M McLaughlin, K D McCloskey, P G Johnston, A Kissenpfennig, D B Longley, C J Scott

**Affiliations:** 1 School of Pharmacy, Queen's University Belfast, Belfast, UK; 2 Centre for Cancer Research and Cell Biology, Queen's University Belfast, Belfast, UK; 3 Department of Physiology, Development and Neuroscience, University of Cambridge, Cambridge, UK; 4 Centre for Infection and Immunity, Queen's University Belfast, Belfast, UK

## Abstract

The simultaneous delivery of multiple cancer drugs in combination therapies to achieve optimal therapeutic effects in patients can be challenging. This study investigated whether co-encapsulation of the BH3-mimetic ABT-737 and the topoisomerase I inhibitor camptothecin (CPT) in PEGylated polymeric nanoparticles (NPs) was a viable strategy for overcoming their clinical limitations and to deliver both compounds at optimal ratios. We found that thrombocytopenia induced by exposure to ABT-737 was diminished through its encapsulation in NPs. Similarly, CPT-associated leukopenia and gastrointestinal toxicity were reduced compared with the administration of free CPT. In addition to the reduction of dose-limiting side effects, the co-encapsulation of both anticancer compounds in a single NP produced synergistic induction of apoptosis in both *in vitro* and *in vivo* colorectal cancer models. This strategy may widen the therapeutic window of these and other drugs and may enhance the clinical efficacy of synergistic drug combinations.

Colorectal cancer is the third most commonly occurring cancer worldwide. Despite advances in understanding the molecular basis of the disease and development of new therapeutic modalities, the 5-year overall survival for patients with late-stage disease remains poor.^1^ Although new compounds are continually developed, the lack of efficacy at systemically tolerable doses frequently precludes their success in the clinic. Formulation and delivery strategies that can improve the narrow therapeutic window of such compounds have the potential to make significant advances in treatment regimens.

ABT-737 is a small molecule that targets the BH3-binding hydrophobic cleft of antiapoptotic B-cell lymphoma (Bcl) proteins Bcl-2, Bcl-w and Bcl-X(L), which are frequently upregulated in tumors and strongly associated with chemoresistance.^2^ As a single agent, ABT-737 is particularly potent against leukaemia/lymphoma cells and a range of small-cell lung carcinomas, causing complete regression of solid tumors in mouse models.^3^ Moreover, its orally bioavailable derivative, ABT-263, has shown potential clinical utility in combination therapies as it has been demonstrated to sensitize tumor types, including colorectal cancer cells, to a range of chemotherapies.^4^ However, the clinical evaluation of ABT-263 has revealed that its therapeutic effects are compromised by severe dose-dependent thrombocytopenia.^5, 6, 7^ Platelets normally survive in circulation for several days by maintaining elevated levels of Bcl-X(L), the inhibition of which by ABT-263/ABT-737 results in premature initiation of platelet apoptosis.^8,9^ In spite of these issues, ABT-263 is still under clinical investigation in combination with frontline cytotoxic chemotherapies such as irinotecan and other therapies (Supplementary Table S1).

Camptothecin (CPT) is a potent topoisomerase-I inhibitor; however, it is poorly soluble and rapidly hydrolyzed from a lactone to an inactive carboxylic acid in an aqueous environment, thus limiting its clinical applicability. Consequently, this has led to the development of derivatives such as irinotecan.^10^ Although irinotecan overcomes some of the pharmacokinetic problems associated with CPT, it has reduced inhibitory activity and still exhibits systemic toxicities such as neutropenia and dose-limiting diarrhea owing to the damage of the intestinal mucosa.^11^

Pharmaceutical formulations that improve the safety profile of potent anticancer drugs such as ABT-737/263 and CPT are urgently required. Drug-loaded nanotherapeutics are finding increasing application in a range of solid tumors, best exemplified by Doxil, a liposomal preparation of doxorubicin that reduces drug-associated myocardiotoxicity.^12^ Such drug carriers can not only diminish adverse effects, but also simultaneously enhance tumor localization through the ability of nanosized particles to penetrate defective endothelial junctions in the tumor neovasculature – a phenomenon known as the enhanced permeability and retention (EPR) effect.^13^ Of note, a polymeric formulation of CPT, CRLX101, has been clinically evaluated, and it was demonstrated that the improved pharmacokinetics and tumor penetration of nanoparticle (NP)-based carriers observed in mouse models were maintained in patients,^14^ highlighting the therapeutic potential that new formulations hold for this parental molecule.

Previously, we developed polymeric NPs encapsulating a range of drug types, formulated in FDA-approved poly-lactide-co-glycolide acid (PLGA).^15, 16, 17^ In the current investigation, we wished to examine the application of both CPT and ABT-737 in colorectal cancer models and determine if a nanodelivery system could be employed to elicit the synergistic efficacies of these agents. We show that nanoencapsulation of ABT-737 reduces thrombocytopenic effects, whereas nanoencapsulation of CPT inhibits its cytotoxic effects towards white blood cells and the gastrointestinal (GI) tract. Both drugs were successfully combined in a single NP formulation to elicit synergistic effects against colorectal cancer cells *in vitro* and *in vivo*; this highlights the potential of this approach and similar formulations for widening narrow therapeutic windows for the treatment of colorectal and other cancers with rationally selected drug combinations delivered at pre-determined synergistic concentrations.

## Results

### Characterization of CPT-loaded NPs

**Figure 1:**
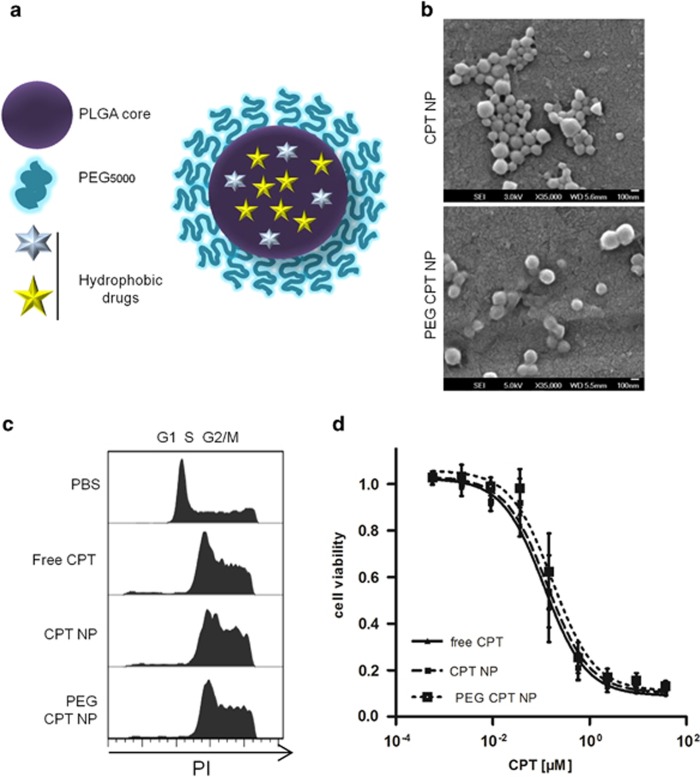
Characterization of CPT-loaded NPs. (**a**) Schematic of drug-loaded PEG-PLGA NPs. (**b**) SEM images of CPT-loaded PLGA and PEG-PLGA NPs, scale bar represents 100 nm. (**c**) Flow cytometric cell cycle analysis (RNase A/PI staining) of murine colorectal MC38 cancer cells following treatment with different CPT formulations (0.12 *μ*M) for 24 h. (**d**) Cell viability of MC38 cells following treatment with different CPT formulations for 48 h was assessed by MTT assay, mean±S.E.M., *n*=3

The objective of this study was to develop a stealth polymeric drug delivery system that would enable the entrapment of CPT and/or ABT-737 within the hydrophobic PLGA core (Figure 1a). Previous work has shown that the introduction of a hydrophilic polyethylene glycol (PEG) corona on the surface of PLGA NPs can enhance their circulation time by reducing protein adsorption and NP uptake by the mononuclear phagocyte system (MPS).^18^ PEG NPs formulated with 25% (w/w) mPEG_5000_-PLGA were of similar size compared with non-PEGylated NPs (Table 1, row 1 and 2, Supplementary Figure S1a), yet elicited reduced *in vitro* macrophage uptake by up to 70% (Supplementary Figure S1b). CPT was encapsulated in both formulations and the NPs were analyzed for size, polydispersity (Table 1, rows 3 and 4) and morphology (Figure 1b), revealing an overall uniform population, with only a minor increase in size and polydispersity compared with non-drug loaded ‘blank' NPs. To ensure that the entrapped drug remained active, the formulations were analyzed for their cytotoxic effects. MC38 murine colorectal cancer cells were incubated with either free CPT or CPT-loaded NPs. Cell cycle analysis after 24 h incubation showed that cells treated with CPT, whether nanoformulated or not, similarly induced S-phase delay and cell cycle arrest in the G2/M phase (Figure 1c). After 48 h, all CPT formulations reduced cell viability in a dose-dependent manner, with comparable EC_50_ for all three formulations of between 0.12 and 0.17 *μ*M (Figure 1d), whereas blank NPs did not impact on cell viability at the highest polymer concentrations (Supplementary Figure S1c). Similar effects with the different CPT formulations were observed against the human colorectal cancer cell line HCT116 (Supplementary Figure S2).

### Nanoencapsulated CPT reduces tumor volume and chemotherapy-associated side effects

**Figure 2:**
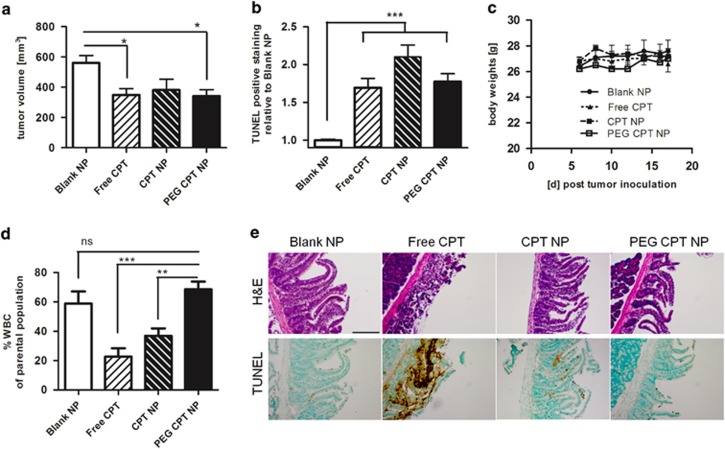
Growth inhibition of syngeneic colorectal tumors and reduction of chemotherapy-associated adverse effects through CPT nanoencapsulation. (**a**) MC38 colorectal tumor volume on day 17 after cell inoculation in C57BL/6 mice and serial treatment with CPT (2.5 mg/kg) on day 6, 10 and 14, mean±S.E.M., *n*≥8. (**b**) Quantification of TUNEL-positive cells analyzed in isolated tumors, mean±S.E.M., *n*=3. (**c**) Mouse body weight during the treatment regime, mean±S.E.M., *n*≥8. (**d**) Quantification of circulating white blood cells (WBC) analyzed by flow cytometry as %WBC of parental population, mean±S.E.M., *n*≥8. (**e**) Images of paraffin-embedded sections of the jejunum stained with H&E or TUNEL after serial CPT treatment, representative scale bar equals 100 *μ*m

To investigate the effects of the different CPT formulations on *in vivo* antitumor activity, equivalent amounts of CPT as free drug, encapsulated in NPs or PEG NPs were administered intravenously on days 6, 10 and 14, respectively, to mice bearing syngeneic MC38 tumors. CPT as free drug and formulated in PEG NPs significantly inhibited the growth of these rapidly growing, aggressive murine tumors by ~30% on day 17; unPEGylated CPT NPs also inhibited tumor growth; however, this failed to reach significance (Figure 2a and Supplementary Figure S3a). These growth-inhibitory effects correlated with enhanced apoptotic events, as the number of TUNEL-stained cells was increased in the tumor tissue for all CPT formulations on day 18 when the study was terminated (Figure 2b and Supplementary Figure S3b). Although all mice appeared healthy without significant weight loss during the study (Figure 2c), the analyses of their blood revealed that free CPT and CPT NPs significantly reduced the numbers of circulating white blood cells, whereas the delivery of CPT in PEG NPs conferred protection against this toxic side effect (Figure 2d and Supplementary Figure S3c). Moreover, there was a clear indication that the nanoencapsulation of CPT was able to reduce the microvilli destruction in the jejunum that was induced by free-drug administration as shown by TUNEL and haematoxylin and eosin (H&E) staining (Figure 2e). Collectively, these data indicate that CPT encapsulated in PEG NPs exhibits similar antitumor effects compared with free drug, but significantly reduces the systemic toxicity of CPT toward white blood cells and the GI tract.

### Nanoencapsulation of ABT-737 reduces thrombocytopenic effects

**Figure 3:**
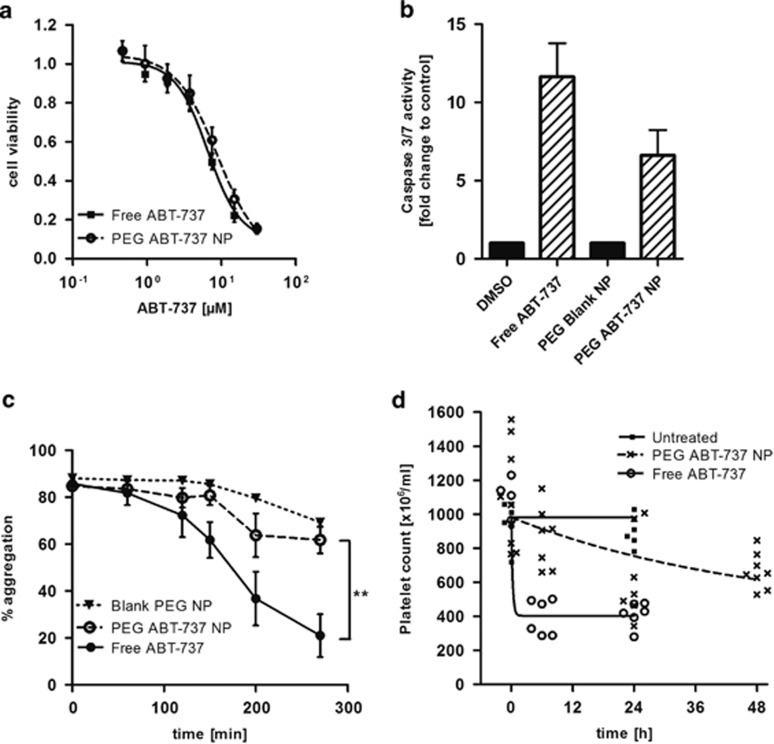
Nanoencapsulation of ABT-737 inhibits thrombocytopenic effects. (**a**) Dose–response curves of HCT116 cells after treatment with free or PEG-PLGA encapsulated ABT-737 for 48 h assessed by MTT cell viability assay, mean±S.E.M., *n*=3. (**b**) Caspase 3/7 activity in HCT116 cells after treatment with free and nanoencapsulated ABT-737 (5 *μ*M) for 6 h, relative to control treated cells, mean±S.E.M., *n*=4. (**c**) Aggregometry of washed human platelets after pre-incubation with free or nanoencapsulated ABT-737 (1 *μ*M), platelet aggregation was induced by U46619 (2 *μ*M), mean±S.E.M., *n*=4. (**d**) Time course of platelet counts in C57BL/6 mice following intraperitoneal injection of 50 mg/kg of free ABT-737 or ABT-737 NPs, *n*≥5. A significant difference was observed between the two ABT-737 treatment groups (*P*<0.0001).The best fit model (highest Schwarz Bayesian Criteria=−398.2) indicated a difference in the rate of change by ~177-fold

The same formulation approach was then used to prepare ABT-737-loaded PEG NPs. ABT-737 is particularly hydrophobic (logP>7) and it was readily entrapped in the NPs, with ‘low drug content' used for initial *in vitro* analysis and, subsequently, ‘high drug content' NPs necessary to achieve therapeutic dosages during *in vivo* assessment (Table 1, rows 5 and 8). The *in vitro* analyses confirmed that the drug was similarly potent against the HCT116 cells whether nanoformulated or not, with an EC_50_ of 6.6±0.9 *μ*M for free ABT-737 and 8.9±1.0 *μ*M for the NP formulation (Figure 3a). Further analysis highlighted that free and encapsulated ABT-737 induced activation of downstream caspases 3 and 7 that was apparent within the first 6 h following treatment, confirming an apoptotic mechanism of cell death (Figure 3b).

We postulated that the encapsulation of ABT-737 in these PEG NPs may protect platelets from its cytotoxic effects. The incubation of human platelets with free ABT-737 induced the inhibition of platelet aggregation over time; in contrast, the encapsulation of ABT-737 in PEG NPs significantly abrogated platelet toxicity *in vitro* (Figure 3c). This effect was then examined *in vivo* as ABT-737 was administered either as a free drug or encapsulated in PEG NPs. Initial platelet counts were no different between the three treatment groups in the model with the highest Schwarz Bayesian Criterion (SBC) (Figure 3d). Both the ABT-737 treatment groups showed a reduction in platelet count, whereas the control group did not. However, the encapsulation of ABT-737 significantly altered the kinetics of the reduction in platelet counts (*P*<0.0001). This effect was best characterized as a 177-fold reduction in the rate constant (*k*) rather than as a change in the extent of the effect. Taken together, these data demonstrate that ABT-737 remains active following nanoformulation, but its platelet toxicity is attenuated as a result of encapsulation.

### Co-encapsulated ABT-737 and CPT exhibit synergistic activity against colorectal cancer cell lines

**Figure 4:**
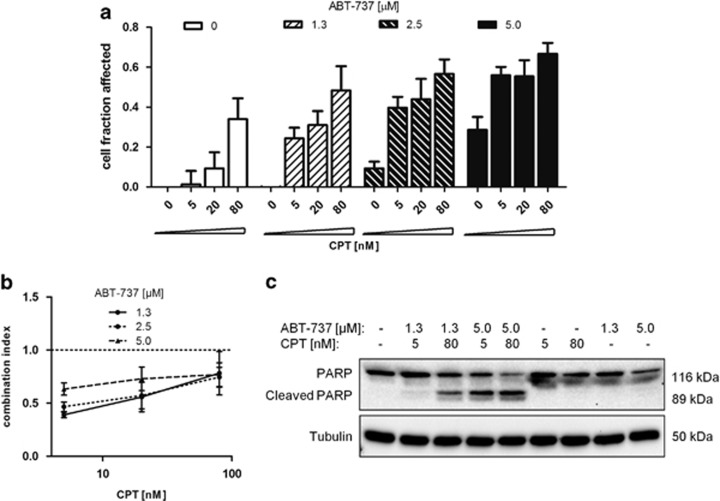
Synergistic effects of free ABT-737 and CPT on colorectal cancer cells. (**a**) Affected (non-viable) cell fractions following treatment of HCT116 cells with ABT-737, CPT and both drugs in combination for 48 h assessed by MTT cell viability assay, mean±S.E.M., *n*=3. (**b**) Combination indices calculated from cell viability data in (**a**), mean±S.E.M., *n*=3. (**c**) Effect of drug combinations on PARP cleavage in HCT116 cells after 24 h incubation

As a single agent, the clinical activity of ABT-263 has been relatively modest, particularly in solid tumors,^5,6^ and therefore its therapeutic utility in combination therapies has been explored. To investigate the combined effectiveness of its parental molecule ABT-737 and CPT, the drugs were co-administered to HCT116 cells, and cytotoxicity determined. Co-incubation with both free drugs revealed synergistic cytotoxic effects, with combination indices of <1 at a broad range of concentrations when ABT-737 was in molar excess to CPT (Figures 4a and b). The drug combinations resulted in markedly enhanced induction of apoptosis as determined by PARP cleavage, which was apparent in ABT-737 and CPT co-treated cells at concentrations at which neither agent alone induced apoptosis (Figure 4c).

**Figure 5:**
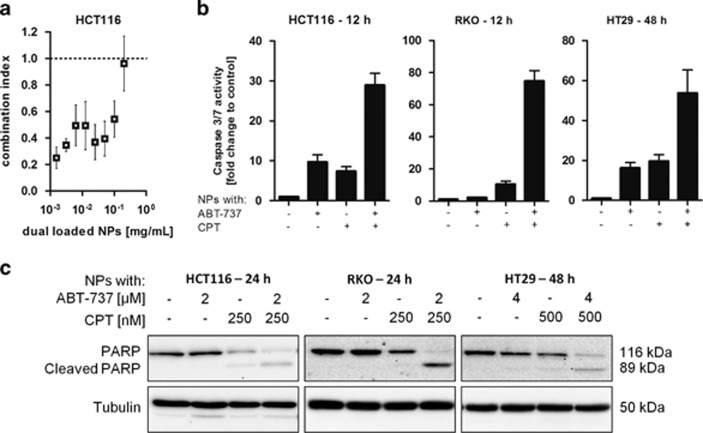
Synergistic effects of ABT-737 on CPT-induced cytotoxicity against colorectal cancer cell lines achieved by co-encapsulation in PEG-PLGA NPs. (**a**) Combination indices obtained from MTT cell viability data following treatment of HCT116 cells with a range of polymer concentrations for 48 h, mean±S.E.M., *n*=3. (**b**) Caspase 3/7 activity following treatment of different colorectal cancer cell lines with ABT-737 (4 *μ*M) or CPT (500 nM) in single- or dual-loaded NPs, mean±S.E.M., *n*≥3. (**c**) Effect of NP formulations on PARP cleavage in HCT116 cells following treatment for 24/48 h

Having established that ABT-737 and CPT produce synergistic proapoptotic effects in HCT116 cells, the potential of translating these effects by generating dual-loaded PEG NP was investigated. Co-encapsulation and control NP formulations were generated for *in vitro* studies with a molar drug ratio CPT:ABT-737 of ~1 : 8 (Table 1, rows 5, 6 and 7). Similar to free drugs, the dual-loaded NPs exhibited synergistic cytotoxic effects toward HCT116 cells at a range of NP concentrations, with CI values as low as 0.3 (Figure 5a). Treatment of HCT116 cells with these NPs resulted in supra-additive increases in caspase 3/7 activity within 12 h of treatment (Figure 5b). This chemosensitizing effect was also revealed by markedly increased PARP cleavage for the dual-loaded particles over single-agent control formulations (Figure 5c). Similar effects were observed in two other colorectal cancer cell lines (Figures 5b and c); the RKO cells showed markedly enhanced caspase 3/7 activity within 12 h of incubation and PARP cleavage at drug concentrations that were ineffective on their own. The HT29 cell line was generally less sensitive to treatment with these agents, and longer incubation (48 h) and higher doses were required to induce a significant apoptotic phenotype.

### Molecular insights into the synergistic action of ABT-737 and CPT

**Figure 6:**
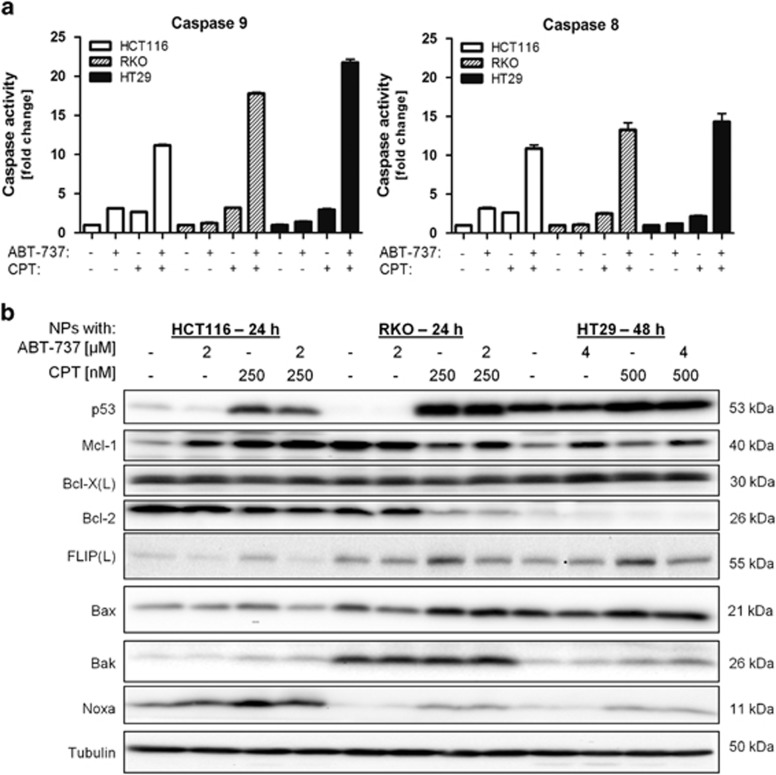
Molecular insights into the synergistic action of ABT-737 and CPT. (**a**) Caspase 9 and caspase 8 activity, mean±S.D., *n*=3, and (**b**) western blot analysis of key pro- and antiapoptotic proteins after treatment with the indicated doses of ABT-737 and CPT in single- or dual-loaded NPs for 24 h (HCT116, RKO) or 48 h (HT29)

In addition to the activation of executioner caspases 3/7 following treatment with dual-loaded NPs (Figure 5), there was also enhanced activation of initiator caspase 9 (indicative of activation of the intrinsic apoptotic pathway) and caspase 8 (indicative of the activation of the extrinsic apoptotic pathway) in all the three cell lines (Figure 6a). By modulating the transcription of a number of pro- and antiapoptotic genes, p53 regulates both the intrinsic and extrinsic apoptotic pathways and was potently upregulated in response to CPT (but not ABT-737) in the two p53 wild-type cell lines HCT116 and RKO (Figure 6b). Interestingly, the R273H mutant p53 expressed in HT29 cells was also upregulated in response to CPT; this common p53 mutant appears to retain some wild-type activity (unpublished observations). Bcl-2 expression was downregulated in response to CPT in both HCT116 and RKO cell lines, whereas Bax was upregulated in all the three cell lines. Another p53-dependent gene, Noxa was also up-regulated in response to CPT. Bcl-X(L) was unchanged in all the treatment groups, whereas Bak expression was moderately upregulated in cells treated with CPT. In addition, both wild-type and mutant forms of p53 can promote apoptosis at a post-transcriptional level by interacting with key proteins of the intrinsic pathway such as Bcl-2, Bcl-X(L), Mcl-1 and Bax.^19,20^ Thus, the increased p53 protein expression induced by CPT may promote activation of proapoptotic Bcl-2 family members, thereby facilitating ABT-737-induced apoptosis.

ABT-737 and ABT-263 do not inhibit Mcl-1, and increased Mcl-1 expression is a major resistance mechanism to ABT-737/263.^21,22^ We found that ABT-737 treatment acutely increased Mcl-1 expression in HCT116 and HT29 cells, but not RKO cells, which already expressed Mcl-1 at high levels (Figure 6b). CPT treatment had varying effects on Mcl-1 expression, with no effect in HT29 cells, increased expression in HCT116 cells and decreased expression in RKO cells. In CPT/ABT-737-co-treated cells, Mcl-1 expression was higher (HCT116 and HT29) or similar (RKO) to untreated cells, suggesting that the mechanism of synergy between CPT and ABT-737 is not dependent on the downregulation of Mcl-1 expression. FLIP is a major regulator of the extrinsic apoptotic pathway through its ability to inhibit death receptor-mediated activation of caspase-8.^23^ Interestingly, CPT treatment at this concentration resulted in increased expression of the major FLIP splice form FLIP(L), and this was attenuated in cells co-treated with CPT and ABT-737 (Figure 6b). We have previously reported that FLIP is a major inhibitor of chemotherapy-induced apoptosis;^24,25^ therefore, attenuation of FLIP(L) upregulation in response to CPT in cells co-treated with ABT-737 is likely to promote caspase-8 activation (Figure 6a) and apoptosis induction.

### Synergistic growth-inhibitory effects in HCT116 xenograft model

**Figure 7:**
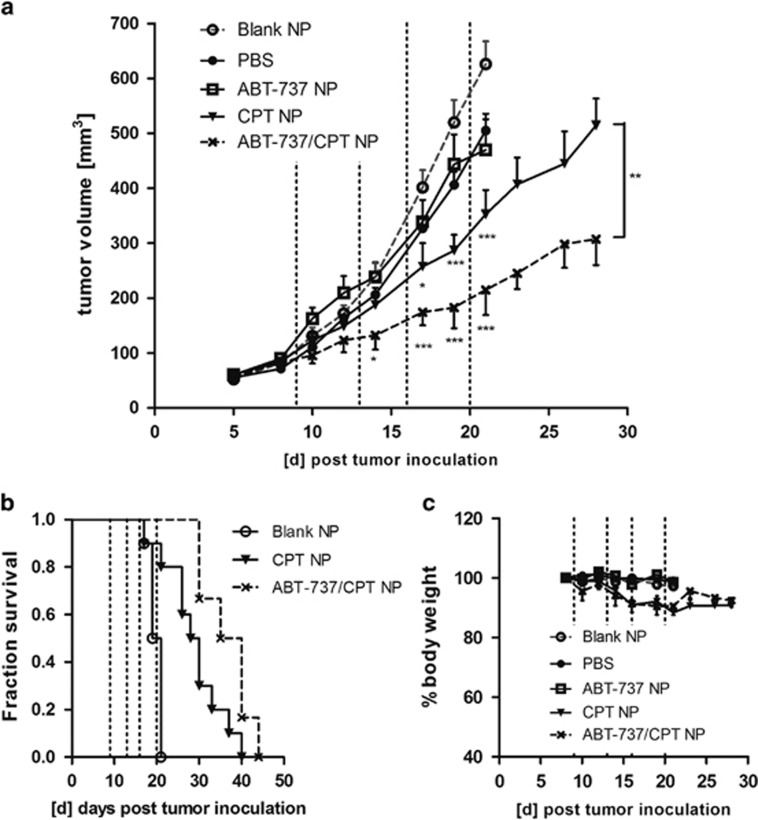
Chemosensitizing effect of ABT-737/CPT dual-loaded NPs against HCT116 xenografts. (**a**) HCT116 xenograft volume over time; the dashed lines indicate the days of treatment with NPs containing 2.1 mg/kg CPT or 50 mg/kg ABT-737, mean±S.E.M., *n*≥6; significance was assessed in comparison to Blank NPs or between the two treatment groups on day 28. (**b**) Kaplan–Meier survival analysis using 500 mm^3^ as the study end point. (**c**) Body weight over the time course, mean±S.E.M., *n*≥3

To achieve appropriate drug doses for *in vivo* evaluation, NP formulations with high drug loading were prepared and analyzed while maintaining the synergistic drug ratio of CPT:ABT-737 close to 1 : 10 (Table 1, rows 8, 9 and 10). These formulations were then used to treat developing HCT116 xenografts. Each drug treatment was given four times between days 8 and 20. Tumor growth analyses revealed that the single-agent ABT-737 NPs did not elicit significant antitumor effects compared with the control arms, whereas mice treated with CPT-only NPs did demonstrate significantly reduced tumor development. Despite its lack of efficacy as a single agent, ABT-737 co-encapsulated with CPT enhanced antitumor activity compared with the CPT-only NPs (Figure 7a). Without any further treatment from day 20, the median survival was enhanced by 8.5 days when 500 mm^3^ tumor volume was used as the study end point for survival analysis (Figure 7b). Although the treatment with CPT-only NPs induced some weight loss (~10%) in the animals, these effects were not severe, and, importantly, despite the enhanced antitumor effects of co-encapsulated CPT and ABT-737, further weight losses were not observed (Figure 7c).

## Discussion

This work demonstrates the ability to reduce toxic side-effects that are associated with the anticancer therapeutics CPT and ABT-737 by encapsulating them in PEGylated polymeric NPs. Furthermore, the combination of both agents in a single NP formulation was found to produce synergistic antitumor effects against a preclinical model of colorectal cancer.

Although both ABT-737/263 and CPT have been superseded by derivatives such ABT-199 and irinotecan, these parental molecules are still under clinical evaluation (Supplementary Table S1),^14^ and, owing to their low water solubility, they are particularly suitable for encapsulation into PLGA-based systems. However, the hydrophobic nature of PLGA can also induce the adsorption of plasma proteins to the NP surface, which promotes their clearance by the MPS.^26^ The incorporation of a hydrophilic PEG corona has previously been found to alleviate this clearance,^27^ and therefore we developed PEG-PLGA NPs with a total of 8% (w/w) PEG. The incorporation of this proportion of PEG sufficiently reduced macrophage uptake, consistent with previous reports identifying an optimal range of 5–10% PEG.^28,29^

Taking this optimized drug carrier formulation forward, we initially considered the potential of the NP to alleviate systemic toxicities associated with our chosen drugs. Dose-limiting side effects of chemotherapies such as leukopenia cause severe challenges for the immune systems of patients.^10,11^ Upon intravenous administration, we found that free CPT reduced the number of white blood cells by up to two-thirds; the number was also significantly reduced by CPT encapsulated in non-PEGylated NPs. However, when administered in PEG NPs, the deleterious effects of CPT on white blood cells were abrogated. This suggests that the ability of the NPs to evade the MPS through PEGylation may have a crucial role in minimizing neutropenic effects. These findings are consistent with recent clinical observations, in which a meta-analysis of nine clinical trials concluded that the occurrence of neutropenia is significantly reduced with liposomal doxorubicin compared with conventionally delivered anthracyclines.^30^ Importantly, we also found clear evidence that encapsulation of CPT in NPs reduces the damage to the GI tract caused by the administration of free drug. GI toxicity found with CPT derivatives is well-established and often requires supportive care in the clinic.^31^ In agreement with our results, the first clinical trials of NP-based delivery of various CPT-derived drugs have reported no or rare grade 3 and 4 diarrhea.^32, 33, 34^

The on-target/off-tumor platelet toxicity of the BH3 mimetic ABT-737 and its analogue ABT-263 has severely limited the clinical utility of these agents.^5, 6, 7^ It has been demonstrated that ABT-737 and ABT-263 reduce the ability of platelets to aggregate by inducing apoptosis, which then results in their rapid clearance.^8,9,35^ This dose-limiting side effect has been shown to be due to the inhibition of Bcl-X(L) and has subsequently led to the development of a Bcl-2-selective inhibitor ABT-199.^36^ However, clinical interest in the inhibition of multiple antiapoptotic proteins including Bcl-X(L) with ABT-263/737 remains. Thus, the encapsulation of such molecules is highly relevant to improve their clinical potential. Here, it was shown that encapsulating ABT-737 into optimized PEG NPs diminished its toxic effects toward platelets *in vitro* and *in vivo*, while maintaining its proapoptotic anticancer effects. Thus, the encapsulation of ABT-737 (or ABT-263) in NPs could potentially circumvent the rapid drop in platelet counts and thereby reduce dose-dependent thrombocytopathy in the clinic, endowing this class of agent with a wider therapeutic window. Moreover, compared with a Bcl-2-selective inhibitor, NP-encapsulated ABT-737 is likely to be more effective against tumors such as colorectal cancer that frequently overexpress Bcl-X(L),^37,38^ the expression of which was uniformly high in the three colorectal cancer models assessed.

On account of the reduced toxicity observed with encapsulated ABT-737 and CPT, both drugs were combined within one formulation to generate a dual cytotoxic strategy. Only a limited number of solid tumors are susceptible to apoptosis following treatment with ABT-737/ABT-263 as a single agent, although it is valued as a chemosensitizer in a range of solid tumors.^3,4^ Synergy was confirmed for both the free drugs in combination and for the dual-loaded NPs, with CI values as low as 0.3. Synergy of ABT-263 with a range of chemotherapies, including CPT, was recently assessed, with average CI values of 0.47, in agreement with our results.^4^

ABT-737 inhibits antiapoptotic Bcl-2, Bcl-X(L) and Bcl-w, thus disrupting their interactions with proapoptotic BH3-only proteins, which then activate Bax and Bak to induce apoptosis.^39,40^ The tumor suppressor protein p53 is stabilized by cell stress such as DNA damage caused by chemotherapy and induces the transcription of proapoptotic Bax and Noxa as well as repressing antiapoptotic Bcl-2,^19,20^ which is in agreement with our observations following CPT treatment. Noxa has been reported to be a critical determinant of ABT-737 sensitivity in solid tumors,^41^ and its induction through treatment with the CPT derivative CPT-11 has been shown to sensitize colorectal cancer cells to ABT-737 . Moreover, it was shown by Okumura *et al.*^39^ that Bax is essential for cytotoxic effects of this combination therapy, and thus the increased levels of Bax expression that we observed in response to CPT may enhance the apoptotic effects of ABT-737. It was also notable that the major inhibitor of the extrinsic apoptotic pathway FLIP was upregulated in response to CPT at this concentration, but this upregulation was abrogated in CPT/ABT-737 co-treated cells. We have previously demonstrated that FLIP is a major inhibitor of chemotherapy-induced apoptosis in colorectal cancer^24,25^ and is frequently overexpressed in this disease.^42^

As a single agent, ABT-737 was shown to be ineffective at inducing significant tumor growth inhibition in several xenograft models of breast, colorectal and ovarian cancer at doses of 50 mg/kg or more; however, it was shown to enhance the antitumor effects of platinum-based chemotherapy and docetaxel.^43, 44, 45^ Here, we observed similar results as the treatment with ABT-737 NPs was ineffective against colorectal xenografts, but synergistically chemo-sensitized the tumors to treatment with CPT. Importantly, the synergistic drug ratio that was established for *in vitro* experiments could successfully be applied to the *in vivo* setting. In conclusion, the work presented herein has shown that the systemic toxicities of CPT and ABT-737 can be reduced by nanoencapsulation and that the two drugs can be combined together in one NP to elicit improved therapeutic effects. This chemosensitizing approach is applicable to combinations of other chemotherapeutics and molecularly targeted agents. Indeed, the co-encapsulation of drug combinations is an area of significant current interest.^46,47^ Importantly, NP formulations can achieve a level of passive targeting of tumors through the EPR effect, enabling the delivery of synergistic drug ratios, thereby overcoming problems caused by differences in the pharmacokinetic profiles of individual agents. Thus, this approach may represent an important advance in current strategies to adminster combination chemotherapies to cancer patients in the clinic.

## Materials and Methods

### NP preparation and characterization

All materials were supplied from Sigma-Aldrich, Dorset, UK unless indicated otherwise. NPs were prepared by single emulsion evaporation using PLGA 502H (Evonik Industries, Essen, Germany) mixed with a 25% (w/w) blend of methoxy PEG_5000_-PLGA (mPEG_5000_-PLGA) copolymer (AK10, 33% PEG content, Akina Inc., West Lafayette, IN, USA). The polymer (20 or 30 mg in total) was dissolved in 1 ml of dichloromethane and added into the aqueous phase containing 2.5% (w/v) polyvinyl alcohol in MES buffer (50 mM, pH 5), sonicated in pulses for 90 s on ice and stirred overnight. ABT-737 (Biorbyt, Cambridge, UK, 65 *μ*g/mg polymer *in vitro*, 333 *μ*g/mg polymer *in vivo*) and/or (S)-(+)-CPT (5 to 33 *μ*g/mg polymer) was added into the organic phase before emulsification. NPs were washed in MES buffer by three wash-spin cycles at 20 000 *g*. ABT-737 and CPT encapsulation was determined by absorbance at 420 nm and fluorescence at 380_Ex_/460_Em_ nm, respectively. Particle size distribution was measured in PBS by dynamic light scattering using a Zetasizer Nano ZS (Malvern Instruments, Worcestershire, UK) and confirmed by scanning electron microscopy (SEM, Jeol 6500 FEGscanning electron microscope, Akishima-Shi, Japan).

### Cell-based assays

The HCT116 cell line was kindly provided by Professor Bert Vogelstein (Johns Hopkins University, Baltimore, MD, USA) and cultured in McCoy medium supplemented with 10% FBS. RKO and HT-29 cells (LGC Standards, Teddington, UK) were cultured in supplemented DMEM. HCT116, HT29 and RKO cell lines were validated by STR profiling by LGC Standards in May 2011. All subsequent cells have only been taken from these validated stocks. The MC38 cell line was a gift from Dr. Steven Rosenberg (National Cancer Institute, NIH) and cultured in supplemented DMEM. All cells were tested regularly for mycoplasma contamination and found to be negative. Cells were seeded into 6-, 12- or 96-well plates and left to adhere overnight. Cell viability was assessed by the addition of MTT reagent (0.5 mg/ml). The resulting formazan crystals were dissolved in DMSO, absorbance measured at 550 nm and cell viability expressed relative to PBS-treated cells. Cell cycle progression was analyzed by flow cytometry (FACSCanto; BD, Oxford, UK). Ethanol-fixed cells were incubated with propidium iodide (PI) and RNase A (Qiagen, Manchester, UK) in PBS/1% FBS for 30 min. Cell lysates were prepared using RIPA lysis buffer containing mini complete protease inhibitors (Roche Applied Science, Penzberg, Germany) and the protein content was determined using the BCA protein Assay Kit (Fisher Scientific, Loughborough, UK). Caspase activity was analyzed in 5 *μ*g of protein using Caspase Glo substrates (Promega, Southampton, UK) and protein expression was assessed by SDS PAGE and western blotting upon semi-dry protein transfer. The membrane was blocked with 5% milk powder in TBS containing 0.1% Tween-20 and incubated with primary antibodies overnight at 4 °C (Noxa, 1 : 250, from Merck Millipore, Feltham, UK; BAX and Bcl-X(L), 1 : 1000 from Cell Signaling Technologies, Danvers, MA, USA; BAK, Bcl-2, 1 : 1000 and p53, 1 : 5000 from Santa Cruz Biotechnology, Heidelberg, Germany; Tubulin, 1 : 10 000 and Mcl-1, 1 : 1000 from Abcam, Cambridge, UK; PARP, 1 : 5000 from eBioscience Ltd., Hatfield, UK; FLIP, 1 : 1000 from Adipogen, Liestal, Switzerland). HRP-conjugated secondary antibody was added for 1 h and the protein visualized by ECL Plus western blotting substrate (Fisher Scientific) using the ChemiDoc XRS+ System (Bio-Rad, Hemel Hempstead, UK).

### Platelet light transmission aggregometry

Ethical approval for the use of blood from human volunteers was granted by the Ethical Committee of the School of Pharmacy, Queen's University Belfast. Human blood was drawn from healthy drug-free volunteers by venepuncture into tri-sodium citrate. Washed platelets were prepared at a concentration of 2 × 10^8^ platelets/ml as previously described.^48^ Four channel platelet aggregation profilers (PAP-4, Bio/Data Corporation, Horsham, PA, USA) were used to assess platelet aggregation, which was induced by U46619 (2 *μ*M). Data are presented as percentage of platelet aggregation obtained from the recorded trace after 6 min.

### *In vivo* experiments

All *in vivo* experiments were carried out in accordance with the Animals (Scientific Procedures) Act, 1986, and approved by the Department of Health, Social Services and Public Safety, Northern Ireland. Mice were purchased from Harlan Laboratories, Bicester, UK, and maintained under controlled environmental conditions (22 °C, 50±10% relative humidity, 12-h light/12-h dark cycle) with food/water *ad libitum* and under sterile conditions when immunocompromised mice were used. A total of 5 × 10^5^ MC38 murine colorectal cancer cells were inoculated subcutaneously in the left flank of male C57BL/6 mice (8–11 weeks old, 27±2 g) using growth factor-reduced Matrigel (BD, diluted to 4 mg/ml in PBS). Mice were randomly assigned into groups and treated intravenously via the tail vein with 2.5 mg/kg CPT or the equivalent amount of CPT-loaded NPs. Free CPT was administrated as a formulation in PBS containing DMSO and PEG400 (14 and 43% (v/v)). The study was terminated on day 18. Blood was withdrawn via cardiac puncture into heparin solution and white blood cell analysis carried out by flow cytometry after red blood cell lysis using OpiLyse B solution (Beckman Coulter Ltd., High Wycombe, UK). The tumors and the GI tract were dissected and fixed in 10% formalin before paraffin-embedding. Sections (6 *μ*m) were stained with Harris H&E (Fisher Scientific) or Terminal deoxynucleotidyl transferase dUTP nick end labeling (TUNEL) using the TumorTACS *in situ* Apoptosis detection kit (Trevigen, Gaithersburg, MD, USA). Images were taken using an Olympus BH2 microscope equipped with DP25 camera and × 10 objective lens, and images were analyzed using Cell B software. TUNEL-positive cells were counted in four fields of view of three tumors of randomly chosen mice.

To analyze the thrombocytopenic effect induced by ABT-737, male C57BL/6 mice (9–12 weeks old, 26±3 g) were injected once intraperitoneally with 50 mg/kg of ABT-737 formulated as previously described^49^ (in 2.5% DMSO, 62.5% H_2_O containing 5% dextrose, 5% Tween-80, 30% propylene glycol, (v/v), final pH 4.5) or equal amounts of ABT-737-loaded NPs in PBS. Before, and 6, 24 and 48 h after the injection, blood was withdrawn using EDTA-coated capillary tubes (Greiner Bio-One, Kremsmünster, Austria) and the platelets were counted with a particle counter (50 *μ*m aperture, Beckman Coulter Ltd.).

To assess synergistic antitumor effects, 2 × 10^6^ HCT116 cells were implanted subcutaneously onto both flanks of female Balb/c nude mice (9–13 weeks, 19±1 g) using Matrigel and the mice were randomly assigned into treatment groups when the tumors reached a volume of ~100 mm^3^. NP formulations were administered intravenously into the tail vein encapsulating equivalents of 2.2 mg/kg CPT and/or 50 mg/kg ABT-737. Body weights were assessed regularly to ensure animal welfare and the tumors were measured every 2–3 days using an electronic calliper calculating the tumor volume according to: 0.5 × length × width^2^.

### Data analysis

Bar and line graphs were plotted with Prism 5.01 software (GraphPad software Inc., La Jolla, CA, USA) and statistical analysis was carried out within. Analysis of variance (ANOVA) with Tukey *post hoc* test was used to investigate statistical differences to compare several groups. The Student's *t*-test was performed for comparison of the two groups. Significant differences are denoted with *P<0.05; **P<0.01; ***P<0.001. Drug synergy was analyzed by following the method of Chou and Talalay.^50^ To investigate thrombocytopenic effects, data were modelled using NONMEM (ICON plc, Dublin, Ireland) to the following structural model of limited exponential decline: 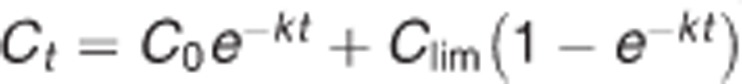, where *t* is the time from administration (h); *C*_*t*_ the platelet count at time *t*; *C*_0_ the platelet count when *t*=0; *C*_lim_ the platelet count at limit of drug effect; *k* the rate constant of decline (h^−1^). Data for the three treatment conditions were fitted simultaneously using nine parameters. Model dimensionality was reduced by constraining parameters and identifying models with the highest Schwarz Bayesian Criterion (SBC=LL—*p* ln(*N*), where LL is the maximized log likelihood, *P* the number of parameters in the model, and *N* the number of data values).^51^ Specific hypotheses were evaluated using a likelihood ratio test.^52^

## Figures and Tables

**Table 1:** Drug loading (per mg of polymer), size and polydispersity index (PDI) of CPT- and/or ABT-737-loaded NPs, mean±S.D., *n*≥4

**Formulation**	**ABT-737 (*****μ*****g/mg)**	**CPT (*****μ*****g/mg)**	**Polymer**	**Size (nm)**^a^	**PDI**^a^
1	—	—	PLGA	181±13	0.06±0.04
2	—	—	PEG-PLGA	160±14	0.07±0.04
3	—	26±5	PLGA	203±19	0.21±0.11
4	—	26±1	PEG-PLGA	187±5	0.14±0.04
5	33±6	—	PEG-PLGA	166±11	0.10±0.07
6	—	2.9±0.3	PEG-PLGA	173±7	0.09±0.09
7	34±6	1.8±0.7	PEG-PLGA	169±12	0.06±0.02
8	263±26	—	PEG-PLGA	212±7	0.11±0.10
9	—	12±2	PEG-PLGA	197±4	0.17±0.05
10	273±17	12±2	PEG-PLGA	212±10	0.12±0.05

a Measured in PBS

## Abbreviations

CPT: camptothecin
PLGA: poly-lactide-co-glycolide acid
PEG: polyethylene glycol
NP: nanoparticle
GI: gastrointestinal
MPS: mononuclear phagocyte system
