# Supplementary material for: Nanoencapsulation of ABT-737 and camptothecin enhances their clinical potential through synergistic antitumor effects and reduction of systemic toxicity
Source: Cell Death Dis. 2014 Oct 9;5(10):e1454–. doi: 10.1038/cddis.2014.413 (PMC4649518; doi:10.1038/cddis.2014.413)
Supplement: Supplementary Information [file cddis2014413x1.pdf]

Supplementary Information

Nano-encapsulation of ABT-737 and camptothecin enhances their clinical potential through synergistic anti-tumor effects and reduction of systemic toxicity

Daniela Schmid [1,2], Gavin E Jarvis [3], Francois Fay [1,5], Donna M Small [4], Michelle K Greene[1], Joanna Majkut [2], Shaun Spence [4], Kirsty M McLaughlin [2], Karen D McCloskey [2], Patrick G Johnston [2], Adrien Kissenpfennig [4], Daniel B Longley [2,†], Christopher J Scott[1,†]

[1] School of Pharmacy, Queen's University Belfast, UK

[2] Centre for Cancer Research and Cell Biology, Queen's University Belfast, UK

[3] Department of Physiology, Development and Neuroscience, University of Cambridge, UK

[4] Centre for Infection and Immunity, Queen's University Belfast, UK

[5] Current address: Translational and Molecular Imaging Institute, Icahn School of Medicine at Mount Sinai, USA

[†] Equal Contribution.

Corresponding author: Professor Christopher Scott
School of Pharmacy
97 Lisburn Road
Queen's University Belfast
BT9 7BL, Belfast, UK
Tel: +44 (0)28 9097 2350
Fax: +44 (0)28 9024 7794
Email: c.scott@qub.ac.uk

This file contains Supplementary Table S1, Supplementary Figures S1-3 and a brief description how nanoparticle uptake was determined for Figure S1b.

Supplementary Table S1

Clinical trials with ABT-263 (Navitoclax).[1]

Phase	Drug(s) in combination	Type of cancer	Trial identifier
I	Ketoconazole	Lymphoid malignancies<br>Solid Tumors	NCT01021358
I	Irinotecan or erlotinib	Solid tumors	NCT01009073
I	Fludarabine,<br>Cyclophosphamide,<br>Rituximab,<br>Bendamustine	Chronic lymphocytic leukemia	NCT00868413
I	Docetaxel	Solid tumors	NCT00888108
I	Rifampin	Solid tumors<br>Lymphoid malignancies	NCT01121133
I	Etoposide, Cisplatin	Solid tumors	NCT00878449
I	Paclitaxel	Solid tumors	NCT00891605
I	Gemcitabine	Solid tumors	NCT00887757
I	Sorafenib tosylate	Relapsed or Refractory Solid Tumors	NCT02143401 [2]
I/II	Rituximab	Lymphoid malignancies<br>Chronic lymphocytic leukemia	NCT00788684<br>NCT01087151
I/II	Dabrafenib, Trametinib	Solid tumors	NCT01989585 [2]<br>NCT02079740 [2]
I/II	Abiraterone,<br>Hydroxychloroquine	Castrate Refractory Prostate Cancer	NCT01828476 [2]
I/II	Trametinib	Solid tumors	NCT02079740

[1] data obtained from *clinicaltrials.gov* (accessed August 2014)
[2] these studies are on-ongoing.

Nanoparticle uptake study

Fluorescent nanoparticles (NP) were generated by addition of 20 µg of rhodamine 6G per 20 mg of polymer and equal dye loading was verified in the NP pellet by measuring fluorescence at $485_{Ex}/520_{Em}$ nm. Raw 264.7 were obtained from ATCC and cultured in supplemented DMEM. Cells were seeded in 6 well plates, adhered overnight and were incubated with 0.1 mg/ml of rhodamine 6G NPs for 1 h. Cells were washed five times in PBS and mean PE fluorescence was analyzed by flow cytometry (Facs Canto, BD).

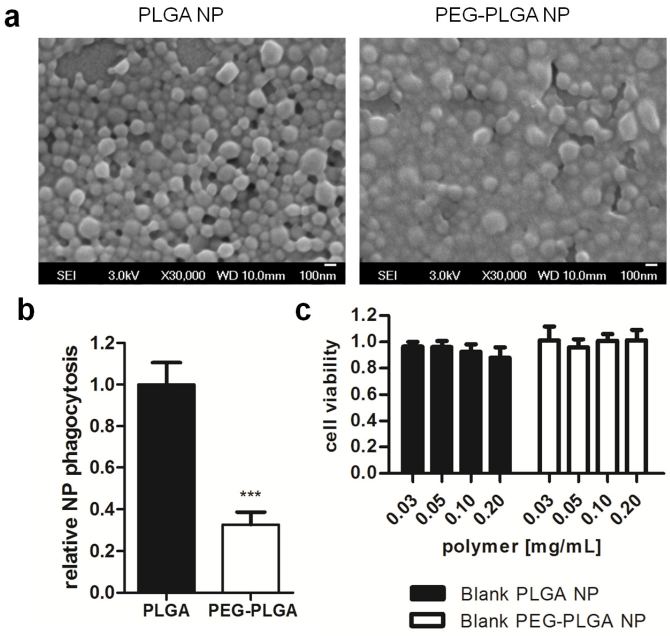

Supplementary Figure S1. Characterization of nanoparticle (NP) formulations.

(a) SEM images of blank PLGA and PEGylated PLGA (PEG-PLGA) NPs confirming uniform size distribution between 100 and 200 nm, scale bar represents 100 nm. (b) Quantification of flow cytometry analysis of Raw 264.7 cells after incubation with 0.1 mg/ml rhodamine 6G labeled PLGA and PEG-PLGA NPs for 1 h, mean±SD, n=3. (c) Cytotoxicity assessment of polymeric NPs against MC38 murine colorectal cancer cells after 48 h analyzed by MTT cell viability assay, mean±SD, n=4.

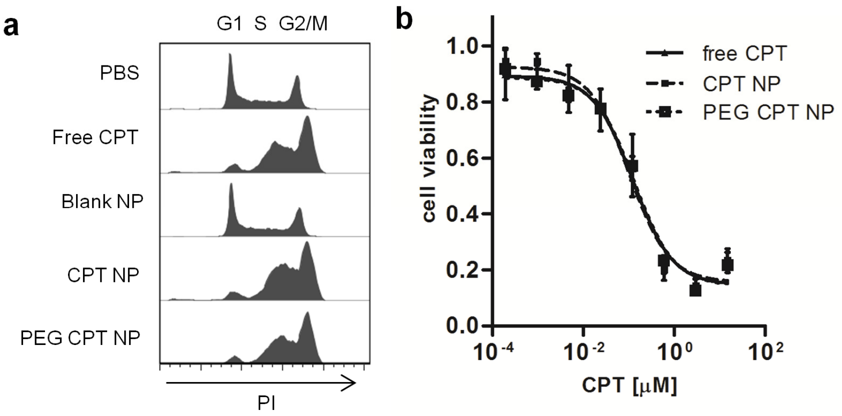

Supplementary Figure S2. Cytotoxic effect of CPT formulations against human HCT116 colorectal cancer cells.

(a) Flow cytometric cell cycle analysis (RNase A/PI staining) following treatment with different CPT formulations (0.12 μM) for 24 h showing S and G2/M arrest. (b) Cell viability following treatment with different CPT formulations for 48 h assessed by MTT cell viability assay, mean±SD, n=3.

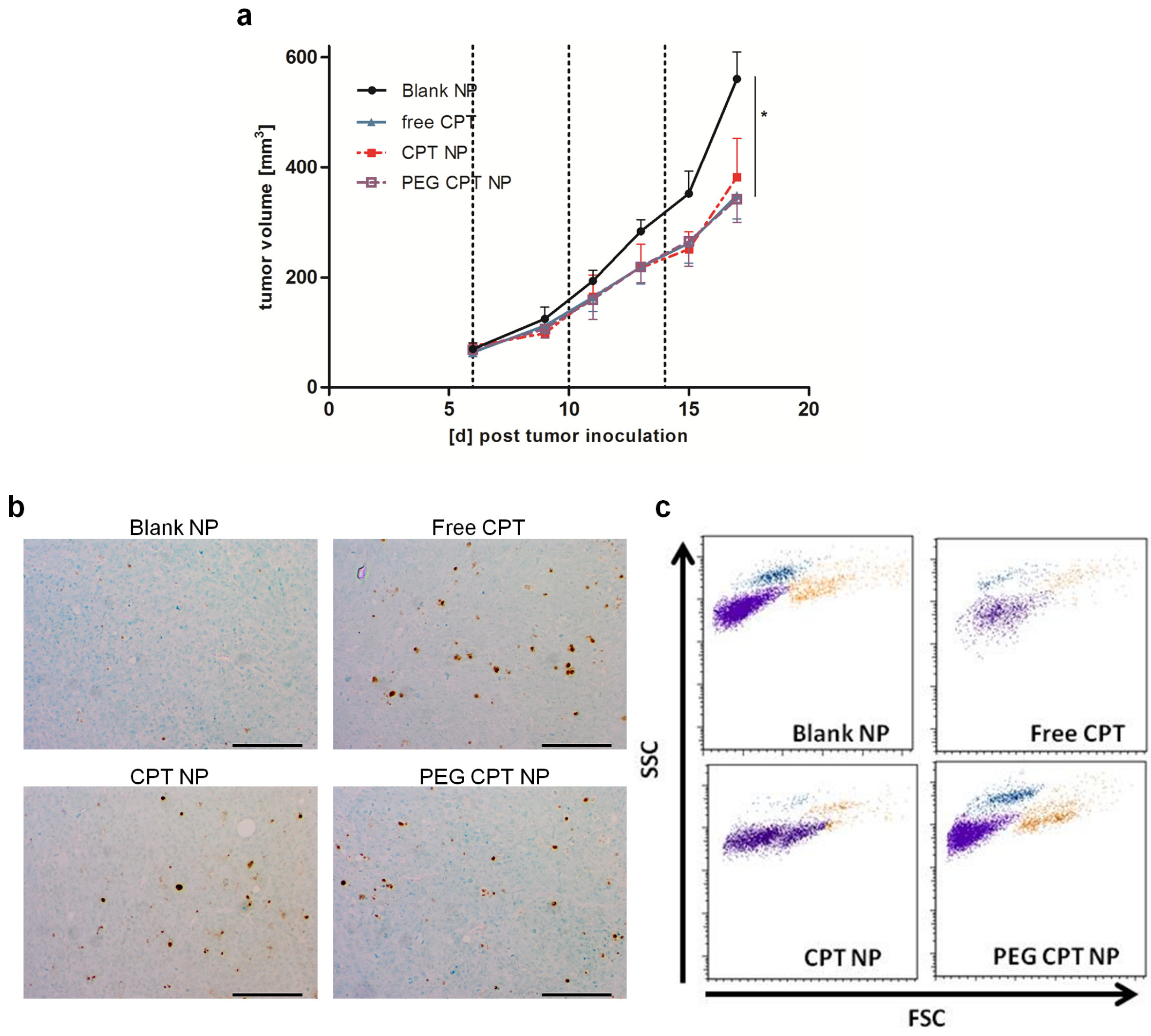

Supplementary Figure S3. Assessment of CPT-induced anti-tumor effect and reduced toxicity.

(a) MC38 colorectal tumor volume over time after cell inoculation in C57BL/6 mice on day 0, the dashed lines indicate the days of treatment with equivalents of 2.5 mg/kg CPT, mean±SEM, n≥8. (b) Representative images of paraffin-embedded sections following TUNEL of tumors dissected on day 18 after three serial treatments as shown in (a); scale bar equals 100 µm. (c) Representative images of circulating white blood cells of C57BL/6 mice analyzed by flow cytometry on day 18 after three serial treatments as shown in (a); lymphocytes presented in purple, granulocytes in orange and monocytes in blue.
